# Athletes With Adductor-Related Groin Pain: A Narrative Review

**DOI:** 10.7759/cureus.68625

**Published:** 2024-09-04

**Authors:** João Dinis, José Ricardo Oliveira, Bárbara Choupina, Pedro Seabra Marques, David Sá, Andre Sarmento

**Affiliations:** 1 Orthopaedics and Traumatology, Unidade Local de Saúde de Gaia/Espinho, Vila Nova de Gaia, PRT

**Keywords:** groin tendon injury, adductor tendinopathy, core muscle injury, pubalgia, adductor-related groin pain

## Abstract

Adductor-related groin pain is extremely common among athletes, and despite its high prevalence and impact, there is no consensus regarding taxonomy, anatomy, physiopathology, or treatment. We performed a comprehensive literature review and tried to demystify this pathology and its treatment. The Doha agreement classification and its impact are scrutinized as well as the complexity of the proximal adductor longus (AL) insertion and its relationship with the pyramidalis-anterior pubic ligament-AL complex. The stress-shielding and compression theories for the origin of AL tendon pathology are exploited along with how this knowledge translates into injury prevention protocols and surgical techniques. The importance of active rehabilitation protocols and intersegmental control-focused programs is highlighted. The role of an enthesis injection in the treatment algorithm is discussed along with when to perform a tenotomy. The differences between selective and complete tenotomy are highlighted.

## Introduction and background

Chronic groin pain is a common complaint among athletes, accounting for up to 15% of all injuries [1]. Among dozens of causes for groin pain, adductor-related pain is the most frequent, being the culprit for up to two-thirds of the cases [2]. In a large cohort of professional soccer players [3], adductor injuries represented 23% of all muscle injuries and were responsible for an average of 14 days of absence from sports.

Although adductor pathology and related groin pain have a high prevalence and impact, they are poorly understood. There is discussion and contradictions in the literature regarding taxonomy, relevant anatomy, pathophysiology, and treatment. The inconsistencies in the taxonomy and the questions surrounding the pathophysiology and anatomy translate not only to the difficulty encountered by the surgeon in identifying the source of pain in the athlete but also the complex anatomy of the region and multiple interactions, including the rectus abdominis insertion, the pyramidalis, the adductor longus (AL) tendon, the gracilis tendon, the anterior pubic ligament, the inguinal canal with its contents, the pubic symphysis, the internal and external oblique, the iliopsoas, and the hip joint. All these structures belong to the same kinetic chain and are under stress when a soccer player kicks a ball, for example. Therefore the same aggression can lead to different injuries, with the involvement of more than one muscle in the pathological process being frequent [2]. One-third of the patients have more than one clinical entity.

Although there are several published systematic reviews [4-9], the discrepancies in diagnostic terms and criteria are so profound that it is nearly impossible to reach a significant conclusion. In this article, we reviewed the available literature in the major databases and tried to identify the controversies surrounding adductor-related groin pain and shed some light on this matter.

## Review

Taxonomy

The terminology for pain in athletes around the groin has been a matter of debate [10]. A systematic review found 33 different diagnostic terms to characterize groin pain [4]. The most frequently used diagnoses were sportsman’s hernia (31%), chronic groin pain (10%), osteitis pubis (10%), adductor-related groin pain (10%), and iliopsoas-related pain (3%, diagnosed as iliopsoas syndrome or iliopsoas tendinitis). Core muscle injury, athletic pubalgia, inguinal disruption, hockey-goalie syndrome, and Gilmore’s groin are also commonly found terms in the literature.

In an attempt to solve this problem, 24 experts from different backgrounds reunited in Doha, Qatar, at the First World Conference on Groin Pain in Athletes in November 2014. Following a Delphi process to achieve agreement, a clinically-based classification was developed [11]. The classification subdivides groin pain into the following three subgroups: the first includes four defined clinical entities for groin pain (adductor-related, iliopsoas-related, inguinal-related, and pubic-related groin pain); the second comprises hip-related groin pain; and the third encompasses other causes of groin pain in athletes.

It is solely based on history and physical examination. During history, the patient should describe pain in the affected region that worsens with exercise. Palpation is the main tool for examination and should be complemented with resistance testing and stretching of the affected muscle groups. Palpation should be precise given the proximity of different structures and is rendered positive if the athlete feels tenderness over the affected area that mimics the usual injury pain. By not adding radiologic criteria, this classification circumvents the uncertainty due to the high prevalence of findings in asymptomatic athletes. In a study by Dallaudiere et al., AL tendon ultrasound abnormalities were found in 45 out of 45 (100%) asymptomatic patients [12]. Loss of normal echogenicity and the fibrillar structure were present in more than 90% of the patients. Further, in a recent Delphi study, experts agreed that clinical evaluation and sport-specific tests should be used to support the return to play (RTP) and not imaging [13].

According to the Doha classification, adductor-related groin pain is defined by a history of pain in the region, adductor tenderness, and pain on resisted adduction testing. This classification has been shown to have excellent interexaminer reliability when the patients presented only one clinical entity but a lower agreement when more than one diagnosis is present [13]. Palpation examination has been found to have a slight to moderate interexaminer reliability while adductor stretch and resisted tests have a moderate-to-substantial correlation [14]. Even though the development of this classification has allowed clearer communication, publications since 2015 have continued to use variable taxonomy, as reported in a systematic review [5].

Relevant anatomy

The proximal insertion of the AL tendon is complex and has been a matter of debate. It was historically considered to be the existence of a continuity of the rectus abdominis (RA) fascia at the pubic insertion with the origin of the AL tendon via an aponeurotic plate [15]. Therefore, these two muscles were considered to act as a pair, counterbalancing each other with the pubis at the center, in a fashion similar to the Copernican theory of the sun [10].

Schilders et al. showed there is no continuity between RA fascia or tendon and AL tendon [16]. AL posteriorly inserts in the pubic bone through a fibrocartilage (1.5 cm × 1.9-2.5 cm) [17,18], and, anteriorly, it is connected to the anterior pubic ligament (APL). This ligament is also the distal insertion of the pyramidalis muscle. The authors named this complex the pyramidalis-anterior pubic ligament-adductor longus complex (PLAC).

A systematic review comprising 76 anatomical cadavers reported similar conclusions [6].​​​ The concept of the aponeurotic plate has become outdated and surgeons should now consider the PLAC. Mathieu et al. recently showed that the gracilis and adductor brevis also have their proximal insertions in the APL and only gracilis and adductor brevis may have an insertion in the inferior pubic ligament [19]. Furthermore, conjoint tendons between these three muscles can be found. The association between gracilis and adductor brevis tendons was the most common (90.9%). Its exact role in the pathophysiology of adductor tendon injuries remains to be elucidated.

The existence of intramuscular tendons in the AL and adductor brevis [17] muscles and the decreasing ratio in the cross-sectional area between the AL tendon and muscle should also be accounted for. Proximally, the ratio of tendon/muscle tissue is approximately 38% to 62%. At 2 cm from the origin, the muscle area comprises approximately 73% [20]. These details help us understand the stress-shielding theory and the role of the selective surgical partial release of the AL tendon.

Of note, in men, the medial RA tendon continues distally with the tendon of the gracilis and fascia lata, while in women, it inserts directly in the anterosuperior aspect of the pubis. The existence of the recto-gracilis tendon in men has gained relevance as it may be one of the reasons behind the increased prevalence of groin pathology in males compared to females [21].

Pathophysiology and prevention

AL tendon injury most commonly affects athletes involved in sports that demand kicking and pivoting frequently, e.g., soccer, hockey, football, and rugby. For instance, in soccer, the AL is under maximal stress when the ipsilateral limb is kicking a ball. Specifically, it is at the greatest risk of injury between 30% and 45% of the swing phase because at this point it is stretching most rapidly while also being eccentrically most active [22]. In these sports, adductor-related groin pain is most commonly caused by an overuse insult. In a cohort study encompassing 2,299 soccer players, adductor-related groin pain most commonly presented a gradual onset (42%) when compared to hamstring (30%), quadriceps (26%), or calf muscle (28%) injuries [3].

Anatomically, the overuse aggression to the AL tendon is considered an insertional tendinopathy. It has been proposed that insertional tendinopathies may have two different pathological mechanisms with the same result: it may be caused by compression or stress-shielding [23,24]. Both mechanisms affect predominantly the deeper part of the tendon. Historically, most surgeons address the AL tendinopathy as an excess of pulling force, i.e., compression mechanism, and therefore perform a complete release of the tendon to decrease this force vector [25,26].

Stress-shielding of the deeper part of the tendon ensues as a consequence of load distribution in the tendon. As the superficial tendon is more distant to the axis of the joint, it is subjected to a higher load while the deeper part is “shielded” from the mechanical stimulus. Having the stress-shielding theory in mind associated with the knowledge of the existence of intramuscular tendons in the AL we have an explanation for the clinical improvement seen with partial release of the AL tendon as only the superficial tendon is cut [27]. The stress-shielding theory also helps us understand the benefit of adductor strengthening in injury prevention. We may hypothesize that strengthening will effectively load the inner part of the tendon.

MRI findings are also consistent with an overuse etiology [28]. Furthermore, enhancement of the anterior pubic region and AL enthesis after gadolinium correlate with the athletes’ symptoms [29]. However, gadolinium-enhanced MRI is not commonly used, and the findings in non-enhanced MRI are also frequently found in asymptomatic individuals.

Assuming its overuse etiology, efforts have been made to identify the risk factors for this injury that could be prevented. Lack of flexibility is considered to be one of the main culprits for suffering an adductor injury. However, in ice hockey players, tight adductors have been shown not to be a risk factor [30]. These findings are aligned with the less successful outcome of stretching and massage rehabilitation programs in chronic adductor injuries when compared with strengthening programs.

A systematic review identified reduced hip abductor and adductor strength, a higher level of play, a previous groin injury, and lower levels of sport-specific training as risk factors for sustaining a groin injury [7]. Regarding specifically adductor injuries, an imbalance between adductor and abductor strength has been recognized as one of the main risk factors. Elite ice hockey players have 17 times higher risk of suffering adductor injury if their adductor strength is 80% or less of the abductor strength [31]. The muscular imbalance between abductor and adductor muscles associated with a decreased adductor strength results in decreased muscle capacity and imbalances during movements in which synergistic function of these groups is necessary as are side-to-side cutting, quick acceleration/deceleration, striding, and sudden direction changes [32,33]. A higher level of play seems to play a role as a risk factor mainly due to the higher intensity of sporting activity and volume [34].

Soccer players who suffer a groin injury have a two times higher risk of suffering a new injury [32], and more than one-third of adductor injuries are recurrent [35]. It is believed previous injury plays a role as a risk factor mainly due to inadequate rehabilitation and healing and specific individual characteristics that place players at greater risk (e.g., anatomical variants, style of play) [36].

Pre-season conditioning and strengthening have been proven to reduce adductor injury rate during the following season [31,37]. The combination of the Copenhagen adduction exercise [38] with other specific exercises has shown the highest efficiency in preventing injuries [37]. The correction of muscle imbalances, the optimization of muscle recruitment, and, therefore, the reduction in muscle fatigue are believed to be the reasons behind its protective role [39].

Conservative treatment

Conservative treatment for longstanding adductor-related groin pain syndrome has not shown reliable and consistent results. A systematic review found only moderate evidence in favor of compression clothing therapy, manual therapy together with strengthening exercise, and prolotherapy. Corticoid injection, platelet-rich plasma therapy, intra-tissue percutaneous electrolysis, and pulse-dose radiofrequency showed conflicting evidence and a lower grade of recommendation [8].

Regarding rehabilitation after injury, the outcome of programs focused on strengthening adductors and pelvic-stabilizing muscles is similar to the findings in pre-season conditioning, being more effective than stretching or local therapy [40]. In a randomized control study of 68 athletes with chronic adductor-related pain, 79% of the patients submitted to active training returned to sports at a median time of 18.5 weeks compared with 14% of the patients who received passive physiotherapy (transcutaneous electrical nerve stimulation, laser treatment, stretching, transverse friction massage). The positive effect of exercise rehabilitation had a long-lasting effect that was still present at 8-12 years after the initial study. The impact was even greater in the subgroup of soccer players [41]. Weir et al. compared a multimodal approach encompassing manual and exercise therapy with isolated exercise therapy and found similar return rates of 50-55%. However, the multimodal group had a shorter time to return [42].

Movement analysis have shown that athletes may present one of three patterns during a 110-degree cutting task: hip, knee, or ankle-dominant [43,44]. Although no association has been reported between a certain pattern and a specific anatomic injury, rehabilitation programs focused on intersegmental control have proven to be superior to programs focused on isolated muscle strength, presenting RTP rates of 73% at a mean time of 9.9 weeks [45].

Overall, it is expected that 20% of the patients will not improve with active rehabilitation. It is essential to promptly recognize these patients to avoid the loss of an extended period of the season by the athlete. Schilders et al. proposed a staging system based on the practice level of the athlete and the presence of enthesopathy in MRI. High-level athletes without abnormal findings on MRI could expect a year free of pain after injection with corticosteroid and topic anesthetic in the AL enthesis; patients of the same level of activity with MRI evidence of enthesopathy could only expect five weeks of relief [46]; in recreational athletes, the infiltration had a 75% success rate, independent of the MRI findings [47]. In all cases, patients were submitted to an active training rehabilitation protocol after the procedure.

Surgical treatment

A systematic review reported that surgical treatment of athletes with groin pain may differ based on the specialty of the surgeon (general surgery or orthopedics), with orthopedic surgeons performing AL tendon tenotomy more frequently [5]. The same study showed that the trend has evolved from performing surgical procedures based on asymptomatic findings to a more tailored approach based on clinical findings. Some surgeons previously advocated for bilateral tenotomy [26] or for an “all-round” approach with abdominal wall repair, neurectomy, and AL tenotomy [48]. In recent studies, more AL tenotomies have been performed.

The evolution in the treatment of these athletes is explained by the increased knowledge of anatomy, and epidemiology and by the experience attained with previous approaches. The importance of the Doha agreement must also be accounted for as it proposed a simple classification to a complex entity, thus providing precise targets to the surgeons.

Regarding patients with adductor-related groin pain, the presence of two theories for tendinopathy, i.e., compression and stress-shielding, gave rise to two surgical approaches and philosophies.

Some studies have advocated for the complete release of the AL tendon at the pubic insertion complemented with a rehabilitation protocol focused on stretching and avoiding reinsertion of the muscle [49,50]. Schilders et al. recommended selective partial adductor release and a rehabilitation protocol focused on adductor muscular strengthening [51].

Gill et al. performed a complete release of the adductor longus tendon in 32 athletes, including 16 National Football League players [49]. Surgery was performed if the patient complained of groin plain below the inguinal ligament, located at the proximal origin of the AL tendon. The pain must have had a duration of more than 10 weeks and must have limited their ability to compete. Schilders et al. performed partial release in 43 professional athletes who complained of AL dysfunction for more than three months and showed no improvement with non-operative treatment. The clinical tests for AL pathology were similar, namely, tenderness over AL insertion and pain with resisted hip adduction. Schilders et al. also considered pain with passive stretch and only considered patients with isolated AL pathology.

The surgical techniques are similar: an incision is made 2 cm below the inguinal crease to avoid maceration, the AL fascia is longitudinally divided, and then the tendon is completely released from the pubic bone (complete tenotomy) or only the superficial part of the tendon is cut 2-4 cm distal to the origin. The rehabilitation protocol follows the surgery. After partial release, the goal is to strengthen the remaining tendon, while after complete release, the goal is to avoid reattachment; hence, strengthening exercises are begun three weeks postoperatively. Both approaches have shown excellent results in high-level athletes with a return rate to the previous level of above 90%. Athletes submitted to partial release took an average of nine weeks to return [51], while athletes in whom complete release was performed needed an average of 12 weeks [49].

Both endoscopic [52] and percutaneous approaches to the tenotomy have been described but have not gained popularity. Endoscopic surgery presents a high level of difficulty and sparse benefits when compared to the 2 cm incision needed for the open approach, and the percutaneous release has shown lower return rates when compared to the open approaches [53].

## Conclusions

Managing an athlete with groin pain is challenging due to the complex anatomy, overlapping clinical presentations, and redundant radiological findings. However, recent advancements have provided valuable tools for surgeons. The Doha agreement offers a straightforward classification based on symptoms and physical examination. Anatomical studies have introduced the PLAC concept, highlighting the attachment of the AL to the APL. Recognizing that AL tendinopathy is an overuse injury associated with stress shielding helps us understand the success of strengthening exercises in prevention, and why a multimodal approach combining manual and exercise therapy is the most effective conservative treatment.

For high-level athletes, MRI can offer prognostic value, with abnormal findings often indicating the need for early surgery. Recreational athletes may experience long-term symptom relief with local infiltrations, regardless of MRI results. Surgery is recommended for athletes with persistent symptoms or high-level athletes with positive MRI findings. AL tenotomy, whether complete or partial, offers similar outcomes, though partial release combined with active rehabilitation may expedite a return to sport. Continued research is essential to standardize diagnostic criteria and management strategies, enabling clearer recommendations.
